# Heart–Gut Axis in Cardiometabolic Disease: Microbiome-Mediated Pathways Linking Metabolic Syndrome to Cardiovascular Risk

**DOI:** 10.3390/medicina62030444

**Published:** 2026-02-26

**Authors:** Tina Bečić, Ivana Jukić, Petra Šimac Prižmić, Ivona Matulić, Hana Đogaš, Mislav Radić, Josipa Radić, Jonatan Vuković, Damir Fabijanić

**Affiliations:** 1 Department of Cardiovascular Diseases, University Hospital of Split, 21000 Split, Croatia; tina.becic@gmail.com (T.B.); damirfabijanic62@gmail.com (D.F.); 2 Department of Internal Medicine, Division of Gastroenterology, University Hospital of Split, 21000 Split, Croatia; 3 Faculty of Health Sciences, University of Split, 21000 Split, Croatia; 4 Department of Internal Medicine, Division of Rheumatology, Allergology and Clinical Immunology, University Hospital of Split, 21000 Split, Croatia; petra_simac@hotmail.com (P.Š.P.); mislavradic@gmail.com (M.R.); 5 Private Clinic Matulic, Osjecka Ulica 24a, 21000 Split, Croatia; ivonamatulic@yahoo.com; 6 Department of Neurology, University Hospital of Split, 21000 Split, Croatia; hana.dogas@gmail.com; 7 Department of Internal Medicine, School of Medicine, University of Split, 21000 Split, Croatia; josiparadic1973@gmail.com; 8 Department of Internal Medicine, Division of Nephrology, Dialysis and Arterial Hypertension, University Hospital of Split, 21000 Split, Croatia; 9 Department of Clinical Propedeutics, School of Medicine, University of Split, 21000 Split, Croatia

**Keywords:** gut microbiota, heart–gut axis, cardiometabolic disease, metabolic syndrome, cardiovascular risk, dysbiosis, microbial metabolites, trimethylamine N-oxide

## Abstract

*Background and Objectives:* Cardiometabolic disease, a term encompassing metabolic syndrome (MS) and cardiovascular disease (CVD), represents a major and growing global health burden driven by interconnected metabolic and cardiovascular dysfunction. Emerging evidence suggests that the gut microbiota plays a central role in modulating metabolic, inflammatory, and cardiovascular (CV) pathways, giving rise to the concept of the heart–gut axis. However, human evidence integrating microbiome-mediated mechanisms across the cardiometabolic spectrum remains incompletely synthesized. This focused systematic review aimed to synthesize the current human evidence on microbiome-mediated mechanisms linking metabolic syndrome (MS) and related metabolic phenotypes with cardiovascular risk (CVR) and subclinical cardiovascular (CV) outcomes within the conceptual framework of the heart–gut axis. *Materials and Methods:* A systematic literature search was conducted in PubMed, Scopus, Web of Science, and the Cochrane Library in accordance with PRISMA 2020 guidelines. Human observational and interventional studies evaluating gut microbiota composition, function, or microbiota-derived metabolites in relation to cardiometabolic, and CV outcomes were included. Risk of bias was assessed using the Cochrane RoB 2 and ROBINS-I tools, and findings were synthesized narratively. *Results*: Ten human studies published between 2016 and 2025 met the inclusion criteria. Across these studies, gut dysbiosis was consistently associated with adverse cardiometabolic risk profiles and subclinical CV outcomes, including insulin resistance, systemic inflammation, subclinical atherosclerosis, and CV prognosis in high-risk populations. Microbiota-derived metabolites, particularly trimethylamine N-oxide (TMAO) and short-chain fatty acids (SCFAs), as well as emerging metabolites such as phenylacetylglutamine (PAGln) and imidazole propionate (ImP), were identified as key mediators linking metabolic syndrome and related metabolic disturbances with CVR and subclinical cardiovascular disease (CVD). Markers of intestinal barrier dysfunction and endotoxemia further supported the role of chronic low-grade inflammation within the heart–gut axis. *Conclusions*: Current human evidence supports the heart–gut axis as a biologically plausible and clinically relevant contributor to cardiometabolic disease. Gut microbiota-derived metabolites, intestinal barrier dysfunction, and systemic inflammation represent interconnected pathways linking MS with CVR. Advancing our understanding of these mechanisms may inform the development of microbiome-targeted strategies to complement established approaches for cardiometabolic and CV prevention.

## 1. Introduction

Cardiometabolic diseases, a term encompassing metabolic syndrome (MS) and cardiovascular disease (CVD), represent a major global health burden and are leading contributors to morbidity and mortality worldwide [1,2]. Although traditionally considered distinct clinical entities, these conditions share common pathophysiological mechanisms, including chronic low-grade inflammation, insulin resistance, endothelial dysfunction, and altered lipid metabolism. In recent years, the gut microbiota has emerged as a central modulator of these interconnected processes, giving rise to the concept of the heart–gut axis [3,4].

The human gut microbiota comprises a complex ecosystem of trillions of microorganisms that interact dynamically with host metabolic, immune, and cardiovascular (CV) systems. Early landmark studies demonstrated that gut microbial composition differs between lean and obese individuals and that the microbiota can directly influence host energy harvest and fat storage [5,6,7]. Subsequent experimental and human studies further established a causal role of the gut microbiota in metabolic regulation, showing that transplantation of microbiota from obese donors can induce metabolic dysfunction in germ-free recipients [8]. These findings positioned gut dysbiosis as a key upstream factor in the development of MS.

In type 2 diabetes mellitus (T2DM), large-scale metagenomic studies have consistently reported disease-specific alterations in gut microbial composition and function, independent of genetic background and geography [9,10]. Importantly, pharmacological treatments such as metformin have been shown to substantially modify the gut microbiome, highlighting the need to disentangle disease-specific microbial signatures from treatment effects [11]. Reviews integrating these findings support a bidirectional relationship between glucose metabolism and the gut microbiota, mediated through inflammation, bile acid signaling, and microbial metabolites [12,13].

Beyond metabolic regulation, growing evidence implicates the gut microbiota in CV pathology. Dysbiosis has been associated with atherosclerosis, hypertension, and heart failure through multiple interrelated mechanisms, including immune activation, endothelial dysfunction, and altered host–microbe metabolic crosstalk [14,15,16]. One of the most extensively studied pathways involves microbiota-derived trimethylamine N-oxide (TMAO), a metabolite produced from dietary phosphatidylcholine and L-carnitine. Elevated circulating TMAO levels have been linked to atherosclerosis, thrombosis, and adverse CV outcomes in both observational and mechanistic studies [17,18,19].

In addition to TMAO, several emerging gut-derived metabolites have been identified as potential mediators of cardiometabolic risk. Phenylacetylglutamine (PAGln) has been shown to promote platelet hyperreactivity and increase cardiovascular risk (CVR) via adrenergic receptor signaling [20]. Imidazole propionate (ImP), a histidine-derived microbial metabolite, impairs insulin signaling through mTORC1 activation and has recently been implicated in both T2DM and atherosclerosis [21,22]. Other microbial products, including short-chain fatty acids (SCFAs) and tryptophan-derived indoles, exert context-dependent effects on inflammation, blood pressure regulation, and metabolic homeostasis [23,24,25].

Despite the rapidly expanding literature, the extent to which gut microbiota–mediated mechanisms integrate MS and CVR within a unified heart–gut axis remains insufficiently synthesized. Existing reviews often focus on single disease entities or individual metabolites, limiting translational interpretation across the cardiometabolic spectrum [26,27]. A comprehensive synthesis of human evidence linking gut dysbiosis, microbial metabolites, and cardiometabolic outcomes is therefore warranted.

Accordingly, this systematic review aims to summarize current human evidence on the role of the gut microbiota in cardiometabolic disease, with a particular focus on MS as key metabolic drivers of CVR. By integrating data on microbial composition, functional pathways, and gut-derived metabolites, this review seeks to clarify mechanistic links within the heart–gut axis and to identify gaps that may inform future microbiome-targeted preventive and therapeutic strategies.

## 2. Materials and Methods

### 2.1. Study Design and Reporting Standards

This systematic review was conducted in accordance with the Preferred Reporting Items for Systematic Reviews and Meta-Analyses (PRISMA) 2020 guidelines [28]. The review protocol was prospectively registered in the International Prospective Register of Systematic Reviews (PROSPERO; ID: CRD420261279340). This review was designed as a focused systematic review to synthesize human evidence on microbiome-mediated mechanisms linking metabolic syndrome with CVR within the heart–gut axis framework. In this context, “focused” refers to an a priori selection strategy prioritizing mechanistic interpretability and translational relevance rather than exhaustive literature coverage. Specifically, inclusion was limited to adult human studies that met three predefined criteria: (i) sufficiently comprehensive gut microbiome characterization, including taxonomic composition with or without functional or metabolite-level analyses; (ii) clearly defined cardiometabolic or CV endpoints; and (iii) direct mechanistic relevance to the heart–gut axis. This intentional trade-off was adopted to reduce conceptual and methodological heterogeneity and to enable a coherent mechanistic synthesis grounded in human evidence. 

Accordingly, this focused approach represents an intentional trade-off between exhaustive literature coverage and conceptual depth, aiming to enhance mechanistic interpretability and translational relevance while maintaining methodological transparency.

### 2.2. Search Strategy

A comprehensive literature search was performed in the following electronic databases: PubMed, Scopus, Web of Science, and the Cochrane Library. The final search was conducted on 21 December 2025. Given the rapidly expanding microbiome literature and the aim to synthesize human evidence specifically addressing microbiome-mediated mechanisms linking MS-related metabolic dysfunction with CVR within the heart–gut axis, we applied a focused (targeted) systematic search strategy. This approach was designed to maximize relevance to mechanistic cardiometabolic pathways (microbiota composition/function, gut barrier integrity, inflammation, and microbiota-derived metabolites) while maintaining feasibility and reproducibility. The search combined terms related to the gut microbiota and dysbiosis with cardiometabolic disease and CVR, explicitly incorporating MS and key cardiometabolic phenotypes (e.g., insulin resistance, hypertension, atherosclerosis, coronary artery disease, and heart failure). To capture mechanistic pathways central to the heart–gut axis, we additionally included terms reflecting intestinal barrier dysfunction, endotoxemia, inflammation, and microbiota-derived metabolites. The final Boolean search string was: (“gut microbiota” OR “gut microbiome” OR dysbiosis) AND (“cardiometabolic disease” OR “cardiovascular risk” OR “cardiovascular disease” OR atherosclerosis OR “coronary artery disease” OR hypertension OR “heart failure”) AND (“metabolic syndrome” OR obesity OR “insulin resistance” OR “type 2 diabetes” OR T2DM) AND (“gut barrier” OR permeability OR endotoxemia OR inflammation OR LPS OR “lipopolysaccharide-binding protein” OR TMAO OR “trimethylamine N-oxide” OR SCFA OR “short-chain fatty acids” OR PAGln OR “phenylacetylglutamine” OR “imidazole propionate” OR indole OR “bile acid”). No restrictions on publication year were applied. Where supported by the database, searches were conducted in titles/abstracts and controlled vocabulary (e.g., MeSH terms) using database-specific adaptations of the strategy. In addition to database searching, the reference lists of all included articles and relevant systematic reviews were manually screened to identify potentially eligible studies not captured through the primary searches.

### 2.3. Eligibility Criteria

#### 2.3.1. Inclusion Criteria

Studies were considered eligible for inclusion if they involved adult human populations and investigated gut microbiota composition, function, or microbiota-derived metabolites in relation to MS, cardiometabolic disease, or CVR. Both observational and interventional study designs were eligible. Included studies were required to report outcomes or mechanistic pathways relevant to the heart–gut axis, such as intestinal barrier dysfunction, endotoxemia, systemic inflammation, or microbiota-derived metabolites, including TMAO.

#### 2.3.2. Exclusion Criteria

Studies were excluded if they were conducted exclusively in animal models or in vitro settings, involved pediatric populations, or lacked adequate characterization of the gut microbiota to support microbiome-related analyses. Publications that did not report relevant cardiometabolic or CV outcomes or did not address mechanistic pathways pertinent to the heart–gut axis, were also excluded. In addition, conference abstracts, editorials, commentaries, and non-systematic narrative reviews were not considered eligible. Studies representing duplicate publications or overlapping populations were excluded to avoid data redundancy. Furthermore, studies were excluded if microbiome analyses were limited to targeted taxa without broader compositional or functional context, or if cardiometabolic outcomes were reported without sufficient methodological detail to allow for meaningful interpretation within the heart–gut axis framework. Where multiple publications reported data from the same cohort, only the most comprehensive or methodologically robust report was included.

### 2.4. Study Selection

**Figure 1:**
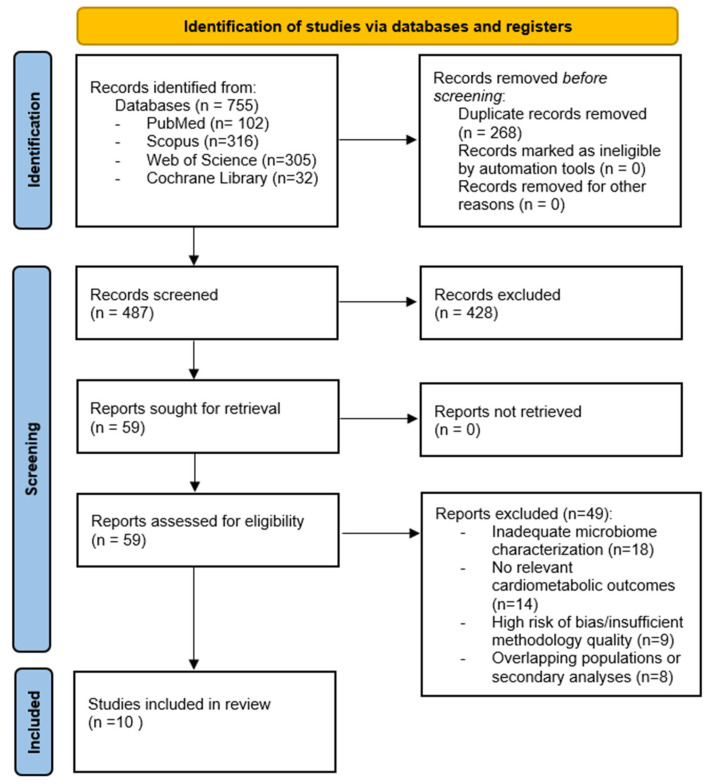
PRISMA 2020 flow diagram of the study selection process for the systematic review. The main reasons for exclusion included inadequate microbiome characterization, lack of relevant cardiometabolic or CV outcomes, insufficient methodological detail for mechanistic interpretation within the heart–gut axis framework, and duplicate or overlapping study populations.

All records identified through database searches were manually reviewed to identify and remove duplicate entries prior to the screening process. Two reviewers (TB and PŠP) independently screened titles and abstracts to assess eligibility. Full-text articles were subsequently retrieved and evaluated in detail according to the predefined inclusion and exclusion criteria. Any discrepancies during the selection process were resolved through discussion and consensus between the reviewers. The study selection process is illustrated in Figure 1 using a PRISMA 2020 flow diagram (Table S1).

### 2.5. Data Extraction

Data extraction was independently performed by two reviewers (TB and PŠP) using a standardized data extraction form. Extracted data included key study characteristics such as the first author and year of publication, study design, sample size and population characteristics, and methods used for microbiome assessment. Information on cardiometabolic and CV outcomes, as well as key findings related to microbiome-mediated mechanisms within the heart–gut axis, was also collected. Any disagreements during data extraction were resolved by consensus.

### 2.6. Risk of Bias Assessment

The risk of bias was independently assessed by two reviewers (TB and PŠP). For randomized controlled trials, the Cochrane Risk of Bias 2.0 tool was used [29], whereas observational studies were evaluated using the ROBINS-I tool [30]. Studies were categorized according to their overall risk of bias. Disagreements between reviewers were resolved through discussion and consensus. Risk of bias assessment was applied exclusively to studies meeting all inclusion criteria and included in the final qualitative synthesis.

### 2.7. Data Synthesis

Given the heterogeneity of study designs, participant characteristics, microbiome assessment methods, and reported outcomes, a quantitative meta-analysis was not considered appropriate. Therefore, a narrative synthesis was conducted. The results were synthesized descriptively and organized according to major microbiome-mediated pathways within the heart–gut axis, including intestinal barrier dysfunction, systemic inflammation, and the role of microbiota-derived metabolites such as TMAO in linking MS with CVR.

## 3. Results

### 3.1. Study Selection

The systematic literature search identified a total of 755 records across four electronic databases, including PubMed, Scopus, Web of Science, and the Cochrane Library. After manual removal of 268 duplicate records, 487 records remained for title and abstract screening. Of these, 428 records were excluded based on predefined eligibility criteria. Fifty-nine full-text articles were subsequently assessed for eligibility. Following full-text evaluation, 49 articles were excluded due to inadequate microbiome characterization, lack of relevant cardiometabolic or CV outcomes, insufficient methodological detail to support microbiome-related analyses, or overlapping populations and secondary analyses. Ultimately, 10 studies met the inclusion criteria and were included in the systematic review. The study selection process is illustrated in Figure 1.

### 3.2. Characteristics of Included Studies

The main characteristics of the studies included in the systematic review are summarized in Table 1.

The final sample comprised 10 human studies published between 2016 and 2025, encompassing a total of observational and interventional designs. Five studies were randomized or interventional clinical trials investigating targeted modulation of the gut microbiota through dietary interventions, probiotics, or pharmacological treatment combined with microbiome-directed strategies. Three studies employed prospective or clinically oriented cohort designs focusing on CV prognosis or subclinical CVD in populations with MS or established coronary artery disease (CAD). The remaining two studies were cross-sectional analyses evaluating associations between gut microbiota composition, microbial metabolites, and cardiometabolic risk factors.

Across the included studies, gut microbiota assessment methods varied and included 16S rRNA gene sequencing and shotgun metagenomic sequencing. Several studies additionally evaluated microbiota-derived metabolites or functional markers, such as SCFAs, TMAO, or lipopolysaccharide (LPS)-binding protein. Cardiometabolic and CV outcomes encompassed measures of insulin sensitivity, glycemic control, lipid profiles, systemic inflammation, blood pressure, subclinical atherosclerosis, and CV prognosis.

### 3.3. Risk of Bias Assessment

Risk of bias was assessed using the RoB 2 tool for randomized controlled trials and interventional studies, and the ROBINS-I tool for observational studies. The results are summarized in Table 2, presented as randomized and interventional studies and observational studies.

Randomized controlled trials were evaluated using the Cochrane Risk of Bias 2.0 (RoB 2) tool [29]. Overall, these studies demonstrated a low risk of bias or some concerns across individual domains, most commonly related to limited reporting of allocation concealment or deviations from intended interventions. No randomized study was judged to be at high risk of bias. Importantly, no study was excluded from the review solely on the basis of risk of bias assessment, as all included studies met the predefined methodological inclusion criteria.

Observational and non-randomized studies were assessed using the ROBINS-I tool [30]. These studies were predominantly judged to have a moderate risk of bias, primarily due to potential residual confounding and, in some cases, participant selection. Bias related to exposure classification, outcome measurement, missing data, and selective reporting was generally assessed as low. Importantly, no study was excluded based on risk of bias assessment alone, as all included studies met the predefined methodological quality thresholds.

### 3.4. Summary of Microbiome-Mediated Cardiometabolic Pathways

Across the included studies, consistent associations were reported between gut microbiota composition or function and cardiometabolic outcomes. Several studies identified alterations in gut microbial diversity or abundance of specific taxa in relation to MS and CVR. Microbiota-derived metabolites, including TMAO and SCFAs, were frequently reported as potential mediators linking gut dysbiosis with metabolic and CV phenotypes. Additionally, markers of intestinal barrier dysfunction and systemic inflammation were assessed in multiple studies, highlighting their relevance within the heart–gut axis.

## 4. Discussion

### 4.1. The Heart–Gut Axis as an Integrative Cardiometabolic Framework

The findings of this systematic review support the heart–gut axis as an integrative biological framework linking MS with CVR. Across the included human studies, alterations in gut microbiota composition, functional potential, and metabolite production were consistently associated with adverse metabolic and cardiovascular phenotypes. These observations reinforce the concept that gut dysbiosis acts as an amplifier of established cardiometabolic risk pathways rather than as an isolated or independent contributor to disease development.

**Figure 2:**
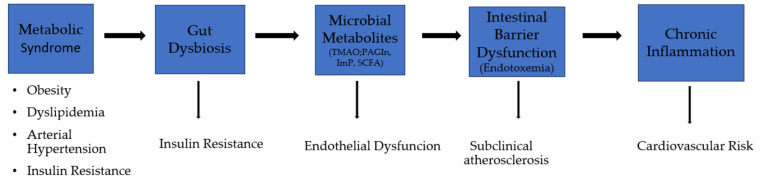
Schematic representation of the heart–gut axis illustrating metabolic syndrome as an upstream amplifier of gut dysbiosis, microbiota-derived metabolites, intestinal barrier dysfunction, and chronic low-grade inflammation. These interconnected pathways contribute to insulin resistance, endothelial dysfunction, subclinical atherosclerosis, and increased cardiovascular risk.

This integrative framework is consistent with prior mechanistic and clinical evidence identifying the gut microbiota as a key interface between environmental exposures and host cardiometabolic regulation [3,4,41]. Diet–microbiota interactions, pharmacological therapies, and lifestyle factors have all been shown to modulate host metabolism and CVR through microbiome-dependent mechanisms [42,43,44]. Collectively, these data support the heart–gut axis as a dynamic, systems-level construct rather than a unidirectional pathway. To facilitate conceptual integration, a graphical summary of the proposed heart–gut axis is provided in Figure 2. The schematic illustrates metabolic syndrome–related dysfunction as an upstream amplifier of gut dysbiosis and microbiome-derived metabolic activity, highlighting key mechanistic nodes—including microbial metabolites, intestinal barrier dysfunction with endotoxemia, and chronic low-grade inflammation—and their downstream cardiometabolic consequences, such as insulin resistance, endothelial dysfunction, subclinical atherosclerosis, and adverse cardiovascular prognosis.

### 4.2. Metabolic Syndrome as Central Driver of Microbiome–Cardiovascular Interactions

MS emerged as central drivers shaping microbiome–CV interactions across the included studies. Both conditions are characterized by insulin resistance, chronic low-grade inflammation, and altered gut microbial ecology, which together create a metabolic milieu that may heighten host susceptibility to microbiome-derived signals. Large-scale metagenomic studies have demonstrated disease-specific alterations in microbial diversity and functional pathways in metabolic syndrome [9,10], findings also supported by population-based microbiome analyses [45].

Importantly, insulin resistance itself represents a key mechanistic link between metabolic disease and CV pathology [46]. Several included studies suggest that metabolic disease states sensitize the CV system to gut-derived metabolites and inflammatory mediators. For example, gut microbiota dysbiosis was associated with adverse CV prognosis in patients with CAD and T2DM [39], while distinct microbial profiles were linked to subclinical CVD in individuals with T2DM [37]. These observations align with experimental data demonstrating that microbiota–lipid interactions can exacerbate adipose tissue inflammation through innate immune signaling pathways [47].

### 4.3. Microbiota-Derived Metabolites: Beyond Trimethylamine N-Oxide

Microbiota-derived metabolites represent a central mechanistic link within the heart–gut axis. TMAO remains the most extensively studied metabolite, with human cohort studies consistently associating elevated circulating TMAO levels with subclinical atherosclerosis, incident CVD, and adverse prognosis [17,18,36,45]. In patients with heart failure, elevated TMAO levels have also been linked to worse clinical outcomes [26,48], underscoring its relevance across the CVD spectrum.

Nevertheless, focusing exclusively on TMAO may oversimplify the complexity of microbiome–cardiometabolic interactions. PAGln has been shown to promote platelet hyperreactivity and increase CVR through adrenergic receptor signaling [20,49]. ImP, a histidine-derived microbial metabolite, impairs insulin signaling via mTORC1 activation and has recently been implicated in both T2DM and atherosclerosis [21,22]. Together, these findings suggest that cardiometabolic risk is shaped by a network of interacting microbial metabolites rather than a single dominant pathway.

### 4.4. Intestinal Barrier Dysfunction, Endotoxemia, and Systemic Inflammation

Disruption of intestinal barrier integrity constitutes an additional key mechanism linking gut dysbiosis with cardiometabolic disease. Several of the included studies evaluated markers of increased intestinal permeability and metabolic endotoxemia, supporting the concept that translocation of microbial components into the systemic circulation contributes to chronic low-grade inflammation. Notably, modulation of the gut microbiota was associated with reductions in LPS-binding protein levels and systemic inflammatory markers in overweight and obese individuals [33].

This inflammatory milieu is a recognized driver of insulin resistance, endothelial dysfunction, and atherosclerosis [12,50]. Experimental and clinical data also support the role of gut barrier dysfunction as a permissive factor that amplifies downstream metabolic and vascular effects of microbiota-derived metabolites [51]. Within the heart–gut axis, impaired intestinal integrity may therefore act synergistically with microbial metabolic activity to promote cardiometabolic disease progression.

### 4.5. Context-Dependent Effects of Short-Chain Fatty Acids and Indole Metabolites

SCFAs exemplify the context-dependent nature of microbiome-mediated cardiometabolic effects. In both experimental models and human studies, SCFAs have been associated with improved insulin sensitivity, lipid metabolism, and blood pressure regulation [23,24]. Emerging evidence further suggests that host sensing of SCFAs through G-protein-coupled receptors may exert protective effects against hypertension [52].

Within the included studies, fecal SCFA profiles reflected underlying cardiometabolic dysbiosis and were associated with obesity and hypertension [38], whereas dietary interventions favorably modulated SCFA patterns in parallel with improvements in metabolic risk markers [34]. In a similar manner, tryptophan-derived indole metabolites, particularly indolepropionic acid, have been associated with a reduced risk of T2DM and related metabolic complications, and lower systemic inflammation [53,54,55]. Collectively, these findings underscore the dualistic and context-dependent roles of microbiome-derived metabolites within the heart–gut axis

### 4.6. Clinical Implications and Translational Potential

The growing recognition of the heart–gut axis has important clinical implications. Microbiome-targeted interventions, including dietary modification, probiotics, fecal microbiota transplantation, and next-generation microbial therapies, have demonstrated potential in improving metabolic parameters in selected populations [56,57,58]. In the interventional studies included in this review, targeted modulation of the gut microbiota improved insulin sensitivity, lipid profiles, and cardiometabolic risk markers [31,32,35]. As summarized in Table 1, the available human evidence spans heterogeneous study designs, including randomized interventional trials, prospective cohorts, and cross-sectional analyses, with CV outcomes predominantly being metabolic and inflammatory markers and subclinical CV phenotypes rather than hard clinical endpoints.

However, interindividual variability in microbiome composition and host response remains substantial. Personalized nutrition and microbiome-informed interventions may therefore be required to achieve clinically meaningful cardiometabolic benefits [59,60]. At present, evidence supporting direct CVR reduction through microbiome-based interventions remains limited, highlighting the need for cautious clinical translation. Importantly, the clinical implications discussed herein should be interpreted primarily as hypothesis-generating. Most of the included studies are observational in nature and rely on surrogate or subclinical cardiovascular outcomes, with a relative paucity of data on hard cardiovascular endpoints. As such, microbiome-targeted strategies should currently be viewed as complementary research avenues rather than established therapeutic interventions for cardiovascular risk reduction.

### 4.7. Methodological Considerations and Future Research Directions

An important methodological consideration in interpreting the available human evidence is the substantial heterogeneity across included studies. Differences in microbiome sequencing techniques (16S rRNA gene sequencing versus shotgun metagenomics), bioinformatic pipelines, dietary exposures, medication use, and population characteristics limit direct comparability and preclude meaningful quantitative synthesis. Rather than attempting to statistically aggregate heterogeneous data, this review adopted a mechanism-oriented narrative approach to contextualize findings within the heart–gut axis framework. As reflected in Table 1, this heterogeneity extends not only to microbiome assessment methods, but also to study design, population risk profiles, and the nature of cardiometabolic and CV outcomes evaluated.

Importantly, heterogeneity was not treated solely as a limitation but as an inherent feature of human microbiome research that informs study selection and interpretation. By prioritizing studies with sufficient microbiome characterization and clinically interpretable cardiometabolic or CV endpoints, the focused design aimed to preserve mechanistic interpretability while acknowledging variability across study designs and populations.

Several methodological considerations warrant careful discussion. The number of eligible human studies remains limited, and substantial heterogeneity is evident across study designs, microbiome assessment techniques, analytical pipelines, and reported cardiometabolic outcomes. Variability in sequencing platforms, bioinformatic workflows, and metabolite quantification methods hampers direct comparability across studies and limits the ability to draw robust quantitative conclusions. Moreover, confounding factors such as dietary patterns, medication use, and lifestyle behaviors are challenging to fully account for, especially in observational study designs, and may substantially influence both microbiome composition and cardiometabolic phenotypes.

Future research should therefore prioritize well-designed longitudinal and interventional human studies that integrate standardized microbiome profiling with complementary multi-omics approaches, including metabolomics, alongside CV imaging and hard clinical endpoints. Harmonization of methodological approaches and reporting standards will be critical to improve reproducibility and cross-study comparability. The application of validated methodological frameworks and quality assessment tools will further strengthen causal inference and enhance the translational relevance of microbiome-based findings within the cardiometabolic field [61,62]. Accordingly, some mechanistic conclusions in this review are derived primarily from smaller or exploratory cohorts, whereas associations involving key microbiota-derived metabolites and subclinical CV phenotypes are supported by larger cohort studies or more consistent findings across independent populations. This distinction should be considered when interpreting the relative strength of the available human evidence. Risk-of-bias assessments further informed the interpretation of findings rather than serving as exclusion criteria. Interventional studies were generally characterized by a low risk of bias or some concerns, whereas observational studies predominantly demonstrated a moderate risk of bias, largely driven by residual confounding and participant selection. These differences were considered when interpreting the strength of evidence, particularly for associations between gut microbiota features and CV outcomes.

### 4.8. Novel Contribution

In contrast to previous reviews that typically focus on isolated components of the gut–heart axis—such as individual metabolites, single disease states, or preclinical evidence—the present review provides an integrated, human-evidence-based synthesis of microbiome-mediated mechanisms linking metabolic syndrome to CVR. A key conceptual contribution is the explicit framing of metabolic syndrome as an amplifying metabolic state that increases host susceptibility to microbiome-derived signals, rather than as a parallel or independent condition. Furthermore, this review uniquely integrates gut microbial composition, functional capacity, microbial metabolites (including TMAO, SCFAs, PAGln and ImP), intestinal barrier dysfunction, and chronic low-grade inflammation into a unified, multilayer mechanistic model grounded exclusively in human studies. By consolidating these interconnected pathways, the review offers a clearer translational framework for understanding how metabolic dysfunction interfaces with CV pathology within the heart–gut axis.

### 4.9. Limitations

The present systematic review has several limitations that should be acknowledged. First, the number of eligible human studies was relatively limited, reflecting the emerging nature of research on the heart–gut axis in cardiometabolic disease. The limited number of eligible studies should be interpreted in the context of the stringent inclusion criteria applied. Only studies with adequate microbiome characterization and clinically interpretable cardiometabolic or CV endpoints were included, which reduced heterogeneity but necessarily limited the size of the final evidence base. This trade-off was considered appropriate to support meaningful mechanistic synthesis rather than broad descriptive aggregation. In addition, substantial heterogeneity was observed across the included studies in terms of study design, population characteristics, microbiome assessment techniques, and reported cardiometabolic and CV outcomes, which limits direct comparability. Second, a substantial proportion of the available evidence was observational, restricting causal inference. Although interventional studies were included, sample sizes were generally modest and follow-up durations were relatively short. Third, variability in microbiome profiling methods and the focus on selected microbial taxa or predefined metabolites may not fully capture the complexity of microbiome-driven cardiometabolic pathways. In addition, the strength of mechanistic inference varies across the included studies. Some conclusions in this review are derived primarily from smaller or exploratory cohorts, whereas associations involving key microbiota-derived metabolites and subclinical CV phenotypes are supported by larger cohorts or more consistent findings across independent populations. This distinction should be considered when interpreting the relative robustness of individual pathways within the heart–gut axis. Despite these limitations, the included studies provide valuable human evidence supporting microbiome-mediated mechanisms linking MS with CVR within the framework of the heart–gut axis.

## 5. Conclusions

This systematic review synthesizes current human evidence supporting the heart–gut axis as a biologically plausible and clinically relevant framework linking MS with CVR. Across both observational and interventional studies, consistent associations were observed between gut dysbiosis, microbiota-derived metabolites, intestinal barrier dysfunction, and adverse cardiometabolic as well as CV outcomes. Collectively, these findings position the gut microbiota as an active modulator of cardiometabolic disease progression rather than a passive bystander. Importantly, this review highlights MS and related metabolic disturbances as central states that may amplify host susceptibility to microbiome-derived signals. Microbial metabolites such as TMAO and SCFAs, together with emerging compounds including PAGln and ImP, appear to operate within interconnected metabolic, inflammatory, and vascular pathways that link gut dysfunction to CV pathology. In parallel, evidence implicating intestinal barrier impairment and low-grade endotoxemia in systemic inflammation further supports a multilayered mechanistic model of the heart–gut axis. From a clinical perspective, the available human data suggest that microbiome-targeted interventions, particularly dietary modulation and selected probiotic strategies, may favorably influence cardiometabolic risk profiles. However, evidence for direct reductions in CVR remains limited, and substantial interindividual variability in baseline microbiome composition and treatment responsiveness continues to represent a major challenge for clinical translation. These observations underscore the need for personalized, context-dependent approaches rather than uniform, one-size-fits-all microbiome interventions. Future research should prioritize well-designed longitudinal and interventional human studies integrating standardized microbiome profiling with metabolomics, CV imaging, and hard clinical endpoints. Such integrative approaches will be essential to strengthen causal inference, identify robust microbial or metabolic biomarkers of CVR, and define patient populations most likely to benefit from microbiome-informed preventive or therapeutic strategies. At present, the principal value of the heart–gut axis lies in its role as a mechanistic and hypothesis-generating framework rather than as a basis for immediate clinical risk stratification or therapeutic decision-making. In conclusion, the heart–gut axis represents a promising conceptual and translational framework for understanding the complex interplay between metabolic dysfunction and CVR. Continued advances in this field may ultimately complement established cardiometabolic prevention strategies and contribute to more integrated, mechanism-based approaches to cardiovascular health.

## Figures and Tables

**Table 1:** Included Studies Evaluating the Heart–Gut Axis and Cardiometabolic Risk.

A. Metabolic Surrogate Markers and Subclinical Cardiovascular Phenotypes						
Study (Author, Year)	Study Design	Population (Sample Size)	Microbiome/Gut-Related Measure	Outcome Category	Key Outcomes	Key Conclusion
Depommier et al., 2019 [31]	Randomized controlled trial	Overweight/obese adults (n = 40)	Akkermansia muciniphila (shotgun metagenomics)	Metabolic surrogate markers	Insulin sensitivity, lipid profile	Targeted microbiota supplementation improved metabolic risk
Reijnders et al., 2016 [32]	Randomized controlled trial	Obese adults (n = 56)	Gut microbiota composition (16S rRNA)	Metabolic surrogate markers	Glucose metabolism, insulin sensitivity	Microbiota manipulation directly affected host metabolism
González-Sarrías et al., 2018 [33]	Randomized clinical trial	Overweight–obese adults (n = 49)	Gut microbiota composition; LPS-binding protein	Metabolic surrogate markers	Systemic inflammation, endotoxemia	Reduced endotoxemia through microbiota modulation
Pagliai et al., 2020 [34]	Dietary intervention study	Overweight adults (n = 107)	Gut microbiota and SCFA profiling	Metabolic surrogate markers	Lipid profile, metabolic risk markers	Mediterranean diet favorably altered microbiota and metabolic risk
Tian J. et al., 2025 [35]	Randomized controlled trial	Patients with T2DM (n = 84)	Gut microbiota (16S rRNA)	Metabolic surrogate markers	Glycemic control, lipids, blood pressure	Probiotics enhanced cardiometabolic benefits of dulaglutide
Randrianarisoa et al., 2016 [36]	Observational cohort study	Adults with cardiometabolic risk (n = 220)	Gut-derived metabolite (TMAO)	Subclinical CV phenotypes	cIMT (early atherosclerosis)	Higher TMAO levels associated with subclinical atherosclerosis
Tsai et al., 2021 [37]	Cross-sectional study	Patients with T2DM (n = 60)	Gut microbiota composition (16S rRNA)	Subclinical CV phenotypes	Subclinical CVD markers	Distinct microbiota profiles linked to subclinical CVD
de la Cuesta-Zuluaga et al., 2019 [38]	Cross-sectional study	Adults with obesity and hypertension (n = 441)	Fecal SCFA and microbiota profiling	Metabolic surrogate markers	Obesity, hypertension	SCFA patterns reflected cardiometabolic dysbiosis
**B. Clinical cardiovascular outcomes and prognosis**						
Study (Author, Year)	Study Design	Population (Sample size)	Microbiome/Gut-Related Measure	Outcome category	Key Outcomes	Key Conclusion
Tian R. et al., 2021 [39]	Prospective cohort study	CAD patients with T2DM (n = 218)	Gut microbiota composition (16S rRNA)	Clinical CV outcomes	Cardiovascular prognosis	Microbiota dysbiosis predicted adverse CV outcomes
Wang et al., 2021 [40]	Prospective cohort study	General adult population (n = 3073)	Shotgun metagenomics	Clinical CV outcomes	Incident cardiometabolic disease	Gut microbiome modulated cardiometabolic protection of Mediterranean diet

**Abbreviations:** CAD, coronary artery disease; cIMT, carotid intima–media thickness; CV, cardiovascular; CVD, cardiovascular disease; LPS, lipopolysaccharide; SCFA, short-chain fatty acid; T2DM, type 2 diabetes mellitus.

**Table 2:** Risk of Bias Assessment of Included Studies.

Study (Author, Year)	Tool	Randomization/Confounding	Selection of Participants	Classification of Exposure	Deviations from Intended Interventions	Missing Data/Outcome Data	Measurement of Outcomes	Selection of Reported Results	Overall Risk of Bias
Depommier et al., 2019 [31]	RoB 2	Low	–	–	Low	Low	Low	Low	Low
Reijnders et al., 2016 [32]	RoB 2	Low	–	–	Some concerns	Low	Low	Low	Some concerns
González-Sarrías et al., 2018 [33]	RoB 2	Low	–	–	Some concerns	Low	Low	Low	Some concerns
Pagliai et al., 2020 [34]	RoB 2	Some concerns	–	–	Some concerns	Low	Low	Low	Some concerns
Tian J. et al., 2025 [35]	RoB 2	Low	–	–	Low	Low	Low	Low	Low
Tian R. et al., 2021 [39]	ROBINS-I	Moderate	Low	Low	Low	Low	Low	Low	Moderate
Randrianarisoa et al., 2016 [36]	ROBINS-I	Moderate	Low	Low	Low	Low	Moderate	Low	Moderate
Tsai et al., 2021 [37]	ROBINS-I	Moderate	Moderate	Low	Low	Low	Moderate	Low	Moderate
de la Cuesta-Zuluaga et al., 2019 [38]	ROBINS-I	Moderate	Moderate	Low	Low	Low	Moderate	Low	Moderate
Wang et al., 2021 [40]	ROBINS-I	Moderate	Low	Low	Low	Low	Low	Low	Moderate

RoB 2, Risk of Bias 2 tool; ROBINS-I, Risk of Bias in Non-randomized Studies of Interventions.

## Data Availability

No new data were created or analyzed in this study. Data sharing is not applicable to this article.

## Supplementary Materials

The following supporting information can be downloaded at: https://www.mdpi.com/article/10.3390/medicina62030444/s1, Table S1: The checklist.

## Abbreviations

The following abbreviations are used in this manuscript:

CAD	Coronary artery disease
cIMT	Carotid intima–media thickness
CV	Cardiovascular
CVR	Cardiovascular risk
CVD	Cardiovascular disease
ImP	Imidazole propionate
LPS	Lipopolysaccharide
MS	Metabolic syndrome
PAGln	Phenylacetylglutamine
PRISMA	Preferred Reporting Items for Systematic Reviews and Meta-Analyses
PROSPERO	International Prospective Register of Systematic Reviews
RCT	Randomized controlled trial
RoB	Risk of bias
ROBINS-I	Risk Of Bias In Non-randomized Studies of Interventions
SCFAs	Short-chain fatty acids
T2DM	Type 2 diabetes mellitus
TMAO	Trimethylamine N-oxide

## References

[1] Libby P. (2002). Inflammation in atherosclerosis. Nature.

[2] Hotamisligil G.S. (2006). Inflammation and metabolic disorders. Nature.

[3] Tang W.H.W., Kitai T., Hazen S.L. (2017). Gut Microbiota in Cardiovascular Health and Disease. Circ. Res..

[4] Brown J.M., Hazen S.L. (2018). Microbial Modulation of Cardiovascular Disease. Nat. Rev. Microbiol..

[5] Bäckhed F., Ding H., Wang T., Hooper L.V., Koh G.Y., Nagy A., Semenkovich C.F., Gordon J.I. (2004). The Gut Microbiota as an Environmental Factor That Regulates Fat Storage. Proc. Natl. Acad. Sci. USA.

[6] Ley R.E., Turnbaugh P.J., Klein S., Gordon J.I. (2006). Microbial Ecology: Human Gut Microbes Associated with Obesity. Nature.

[7] Turnbaugh P.J., Ley R.E., Mahowald M.A., Magrini V., Mardis E.R., Gordon J.I. (2006). An Obesity-Associated Gut Microbiome with Increased Capacity for Energy Harvest. Nature.

[8] Ridaura V.K., Faith J.J., Rey F.E., Cheng J., Duncan A.E., Kau A.L., Griffin N.W., Lombard V., Henrissat B., Bain J.R. (2013). Gut Microbiota from Twins Discordant for Obesity Modulate Metabolism in Mice. Science.

[9] Qin J., Li Y., Cai Z., Li S., Zhu J., Zhang F., Liang S., Zhang W., Guan Y., Shen D. (2012). A Metagenome-Wide Association Study of Gut Microbiota in Type 2 Diabetes. Nature.

[10] Karlsson F.H., Tremaroli V., Nookaew I., Bergström G., Behre C.J., Fagerberg B., Nielsen J., Bäckhed F. (2013). Gut Metagenome in European Women with Normal, Impaired and Diabetic Glucose Control. Nature.

[11] Forslund K., Hildebrand F., Nielsen T., Falony G., Le Chatelier E., Sunagawa S., Prifti E., Vieira-Silva S., Gudmundsdottir V., Pedersen H.K. (2015). Disentangling Type 2 Diabetes and Metformin Treatment Signatures in the Human Gut Microbiota. Nature.

[12] Tilg H., Moschen A.R. (2014). Microbiota and Diabetes: An Evolving Relationship. Gut.

[13] Gurung M., Li Z., You H., Rodrigues R., Jump D.B., Morgun A., Shulzhenko N. (2020). Role of Gut Microbiota in Type 2 Diabetes Pathophysiology. EBioMedicine.

[14] Jonsson A.L., Bäckhed F. (2017). Role of Gut Microbiota in Atherosclerosis. Nat. Rev. Cardiol..

[15] Witkowski M., Weeks T.L., Hazen S.L. (2020). Gut Microbiota and Cardiovascular Disease. Circ. Res..

[16] Yamashita T. (2025). The Role of Gut Microbiota in Cardiovascular Diseases and Their Potential as Novel Therapeutic Targets. J. Cardiol..

[17] Wang Z., Klipfell E., Bennett B.J., Koeth R., Levison B.S., Dugar B., Feldstein A.E., Britt E.B., Fu X., Chung Y.-M. (2011). Gut Flora Metabolism of Phosphatidylcholine Promotes Cardiovascular Disease. Nature.

[18] Koeth R.A., Wang Z., Levison B.S., Buffa J.A., Org E., Sheehy B.T., Britt E.B., Fu X., Wu Y., Li L. (2013). Intestinal Microbiota Metabolism of L-Carnitine, a Nutrient in Red Meat, Promotes Atherosclerosis. Nat. Med..

[19] Tang W.H.W., Wang Z., Levison B.S., Koeth R.A., Britt E.B., Fu X., Wu Y., Hazen S.L. (2013). Intestinal Microbial Metabolism of Phosphatidylcholine and Cardiovascular Risk. N. Engl. J. Med..

[20] Nemet I., Saha P.P., Gupta N., Zhu W., Romano K.A., Skye S.M., Cajka T., Mohan M.L., Li L., Wu Y. (2020). A Cardiovascular Disease-Linked Gut Microbial Metabolite Acts via Adrenergic Receptors. Cell.

[21] Koh A., Molinaro A., Ståhlman M., Khan M.T., Schmidt C., Mannerås-Holm L., Wu H., Carreras A., Jeong H., Olofsson L.E. (2018). Microbially Produced Imidazole Propionate Impairs Insulin Signaling through mTORC1. Cell.

[22] Mastrangelo A., Robles-Vera I., Mañanes D., Galán M., Femenía-Muiña M., Redondo-Urzainqui A., Barrero-Rodríguez R., Papaioannou E., Amores-Iniesta J., Devesa A. (2025). Imidazole propionate is a driver and therapeutic target in atherosclerosis. Nature.

[23] Canfora E.E., Meex R.C.R., Venema K., Blaak E.E. (2019). Gut microbial metabolites in obesity, NAFLD and T2DM. Nat. Rev. Endocrinol..

[24] Koh A., De Vadder F., Kovatcheva-Datchary P., Bäckhed F. (2016). From Dietary Fiber to Host Physiology: Short-Chain Fatty Acids as Key Bacterial Metabolites. Cell.

[25] Xu J., Moore B.N., Pluznick J.L. (2022). Short-Chain Fatty Acid Receptors and Blood Pressure Regulation: Council on Hypertension Mid-Career Award for Research Excellence 2021. Hypertension.

[26] Tang W.H.W., Li D.Y., Hazen S.L. (2019). Dietary Metabolism, the Gut Microbiome, and Heart Failure. Nat. Rev. Cardiol..

[27] Masenga S.K., Hamooya B., Hangoma J., Hayumbu V., Ertuglu L.A., Ishimwe J., Rahman S., Saleem M., Laffer C.L., Elijovich F. (2022). Recent Advances in Modulation of Cardiovascular Diseases by the Gut Microbiota. J. Hum. Hypertens..

[28] Page M.J., McKenzie J.E., Bossuyt P.M., Boutron I., Hoffmann T.C., Mulrow C.D., Shamseer L., Tetzlaff J.M., Akl E.A., Brennan S.E. (2021). The PRISMA 2020 statement: An updated guideline for reporting systematic reviews. BMJ.

[29] Sterne J.A.C., Savović J., Page M.J., Elbers R.G., Blencowe N.S., Boutron I., Cates C.J., Cheng H.-Y., Corbett M.S., Eldridge S.M. (2019). RoB 2: A revised tool for assessing risk of bias in randomised trials. BMJ.

[30] Sterne J.A.C., Hernán M.A., Reeves B.C., Savović J., Berkman N.D., Viswanathan M., Henry D., Altman D.G., Ansari M.T., Boutron I. (2016). ROBINS-I: A tool for assessing risk of bias in non-randomised studies of interventions. BMJ.

[31] Depommier C., Everard A., Druart C., Plovier H., Van Hul M., Vieira-Silva S., Falony G., Raes J., Maiter D., Delzenne N.M. (2019). Supplementation with *Akkermansia muciniphila* in overweight and obese human volunteers: A proof-of-concept exploratory study. Nat. Med..

[32] Reijnders D., Goossens G.H., Hermes G.D.A., Neis E.P.J.G., van der Beek C.M., Most J., Holst J.J., Lenaerts K., Kootte R.S., Nieuwdorp M. (2016). Effects of Gut Microbiota Manipulation by Antibiotics on Host Metabolism in Obese Humans: A Randomized Double-Blind Placebo-Controlled Trial. Cell Metab..

[33] González-Sarrías A., Romo-Vaquero M., García-Villalba R., Cortés-Martín A., Selma M.V., Espín J.C. (2018). The Endotoxemia Marker Lipopolysaccharide-Binding Protein Is Reduced in Overweight-Obese Subjects Consuming Pomegranate Extract by Modulating the Gut Microbiota: A Randomized Clinical Trial. Mol. Nutr. Food Res..

[34] Pagliai G., Russo E., Niccolai E., Dinu M., Di Pilato V., Magrini A., Bartolucci G., Baldi S., Menicatti M., Giusti B. (2020). Influence of a 3-Month Low-Calorie Mediterranean Diet Compared to the Vegetarian Diet on Human Gut Microbiota and SCFA: The CARDIVEG Study. Eur. J. Nutr..

[35] Tian J., Yang Y., Yu Z., Gao Y., Zong X., Wu Q., Su H., Cao W., Xu D. (2025). Comparative Evaluation of Dulaglutide Alone vs. Dulaglutide Combined with Probiotics on Cardiovascular Risk Factors in T2DM. Hormones.

[36] Randrianarisoa E., Lehn-Stefan A., Wang X., Hoene M., Peter A., Heinzmann S.S., Zhao X., Königsrainer I., Königsrainer A., Balletshofer B. (2016). Relationship of Serum Trimethylamine N-Oxide (TMAO) Levels with Early Atherosclerosis in Humans. Sci. Rep..

[37] Tsai H.-J., Tsai W.-C., Hung W.-C., Hung W.-W., Chang C.-C., Dai C.-Y., Tsai Y.-C. (2021). Gut Microbiota and Subclinical Cardiovascular Disease in Patients with Type 2 Diabetes Mellitus. Nutrients.

[38] de la Cuesta-Zuluaga J., Mueller N.T., Álvarez-Quintero R., Velásquez-Mejía E.P., Sierra J.A., Corrales-Agudelo V., Carmona J.A., Abad J.M., Escobar J.S. (2019). Higher Fecal Short-Chain Fatty Acid Levels Are Associated with Gut Microbiome Dysbiosis, Obesity, Hypertension and Cardiometabolic Disease Risk Factors. Nutrients.

[39] Tian R., Liu H., Feng S., Wang H., Wang Y., Wang Y., Liang L., Xu H., Xing H., Zhang S. (2021). Gut Microbiota Dysbiosis in Stable Coronary Artery Disease Combined with Type 2 Diabetes Mellitus Influences Cardiovascular Prognosis. Nutr. Metab. Cardiovasc. Dis..

[40] Wang D.D., Nguyen L.H., Li Y., Yan Y., Ma W., Rinott E., Ivey K.L., Shai I., Willett W.C., Hu F.B. (2021). The Gut Microbiome Modulates the Protective Association between a Mediterranean Diet and Cardiometabolic Disease Risk. Nat. Med..

[41] Tremaroli V., Bäckhed F. (2012). Functional interactions between the gut microbiota and host metabolism. Nature.

[42] Sonnenburg J.L., Bäckhed F. (2016). Diet–microbiota interactions as moderators of human metabolism. Nature.

[43] David L.A., Maurice C.F., Carmody R.N., Gootenberg D.B., Button J.E., Wolfe B.E., Ling A.V., Devlin A.S., Varma Y., Fischbach M.A. (2014). Diet rapidly and reproducibly alters the human gut microbiome. Nature.

[44] Wu G.D., Chen J., Hoffmann C., Bittinger K., Chen Y.-Y., Keilbaugh S.A., Bewtra M., Knights D., Walters W.A., Knight R. (2011). Linking long-term dietary patterns with gut microbial enterotypes. Science.

[45] Jie Z., Xia H., Zhong S.-L., Feng Q., Li S., Liang S., Zhong H., Liu Z., Gao Y., Zhao H. (2017). The Gut Microbiome in Atherosclerotic Cardiovascular Disease. Nat. Commun..

[46] Boucher J., Kleinridders A., Kahn C.R. (2014). Insulin receptor signaling in normal and insulin resistant states. Cold Spring Harb. Perspect. Biol..

[47] Caesar R., Tremaroli V., Kovatcheva-Datchary P., Cani P.D., Bäckhed F. (2015). Crosstalk between Gut Microbiota and Dietary Lipids Aggravates WAT Inflammation through TLR Signaling. Cell Metab..

[48] Li X., Fan Z., Cui J., Li D., Lu J., Cui X., Xie L., Wu Y., Lin Q., Li Y. (2022). Trimethylamine N-Oxide in Heart Failure: A Meta-Analysis of Prognostic Value. Front. Cardiovasc. Med..

[49] Ottosson F., Brunkwall L., Smith E., Orho-Melander M., Nilsson P.M., Fernández C., Melander O. (2020). The gut microbiota-related metabolite phenylacetylglutamine associates with increased risk of incident coronary artery disease. J. Hypertens..

[50] Cani P.D., Amar J., Iglesias M.A., Poggi M., Knauf C., Bastelica D., Neyrinck A.M., Fava F., Tuohy K.M., Chabo C. (2007). Metabolic Endotoxemia Initiates Obesity and Insulin Resistance. Diabetes.

[51] Lynch S.V., Pedersen O. (2016). The human intestinal microbiome in health and disease. N. Engl. J. Med..

[52] Muralitharan R.R., Zheng T., Dinakis E., Xie L., Barbaro-Wahl A., Jama H.A., Nakai M., Paterson M., Leung K.C., McArdle Z. (2025). Gut Microbiota Metabolites Sensed by Host GPR41/43 Protect Against Hypertension. Circ. Res..

[53] de Mello V.D., Paananen J., Lindström J., Lankinen M.A., Shi L., Kuusisto J., Pihlajamäki J., Auriola S., Lehtonen M., Rolandsson O. (2017). Indolepropionic acid and novel lipid metabolites are associated with a lower risk of type 2 diabetes in the Finnish Diabetes Prevention Study. Sci. Rep..

[54] Tuomainen M., Lindström J., Lehtonen M., Auriola S., Pihlajamäki J., Peltonen M., Tuomilehto J., Uusitupa M., de Mello V.D., Hanhineva K. (2018). Associations of serum indolepropionic acid, a gut microbiota metabolite, with type 2 diabetes and low-grade inflammation in high-risk individuals. Nutr. Diabetes.

[55] Sehgal R., de Mello V.D., Männistö V., Lindström J., Tuomilehto J., Pihlajamäki J., Uusitupa M. (2022). Indolepropionic Acid, a Gut Bacteria-Produced Tryptophan Metabolite and the Risk of Type 2 Diabetes and Non-Alcoholic Fatty Liver Disease. Nutrients.

[56] Vrieze A., Van Nood E., Holleman F., Salojärvi J., Kootte R.S., Bartelsman J.F.W.M., Dallinga-Thie G.M., Ackermans M.T., Serlie M.J., Oozeer R. (2012). Transfer of intestinal microbiota from lean donors increases insulin sensitivity in individuals with metabolic syndrome. Gastroenterology.

[57] de Groot P.F., Frissen M.N., de Clercq N.C., Nieuwdorp M. (2017). Fecal microbiota transplantation in metabolic syndrome: History, present and future. Gut Microbes.

[58] Zmora N., Zilberman-Schapira G., Suez J., Mor U., Dori-Bachash M., Bashiardes S., Kotler E., Zur M., Regev-Lehavi D., Ben-Zeev Brik R. (2018). Personalized Gut Mucosal Colonization Resistance to Empiric Probiotics Is Associated with Unique Host and Microbiome Features. Cell.

[59] Zeevi D., Korem T., Zmora N., Israeli D., Rothschild D., Weinberger A., Ben-Yacov O., Lador D., Avnit-Sagi T., Lotan-Pompan M. (2015). Personalized nutrition by prediction of glycemic responses. Cell.

[60] Zhang Y., Liu R., Chen Y., Cao Z., Liu C., Bao R., Wang Y., Huang S., Pan S., Qin L. (2025). *Akkermansia muciniphila* supplementation in patients with overweight/obese type 2 diabetes: Efficacy depends on its baseline levels in the gut. Cell Metab..

[61] Shea B.J., Reeves B.C., Wells G., Thuku M., Hamel C., Moran J., Moher D., Tugwell P., Welch V., Kristjansson E. (2017). AMSTAR 2: A critical appraisal tool for systematic reviews that include randomised or non-randomised studies of healthcare interventions, or both. BMJ.

[62] Guyatt G.H., Oxman A.D., Vist G.E., Kunz R., Falck-Ytter Y., Alonso-Coello P., Schünemann H.J. (2008). GRADE Working Group. GRADE: An emerging consensus on rating quality of evidence and strength of recommendations. BMJ.

